# Gut microbiota–host co-metabolism in sepsis and critical illness: source, compartment, immune state, and host defense

**DOI:** 10.3389/fmicb.2026.1975307

**Published:** 2026-10-26

**Authors:** Chongchong Wu, Rui Liu

**Affiliations:** Department of Emergency Medicine, The Second Affiliated Hospital of Zhejiang Chinese Medical University, Hangzhou, China

**Keywords:** critical illness, gut microbiota, host–microbiota interactions, immune reprogramming, immunometabolism, microbial–host co-metabolism, sepsis, source attribution

## Abstract

Critical illness can profoundly disturb the intestinal microbiota–metabolite–host immune axis through the combined effects of shock, hypoxia, altered nutritional intake, antimicrobial exposure, organ dysfunction, and impaired epithelial barrier integrity. These changes can reshape microbial metabolic capacity, host co-metabolism, and systemic metabolite exposure, while the host may simultaneously exhibit overlapping features of hyperinflammation, immune tolerance, lymphocyte dysfunction, impaired antimicrobial defense, and tissue repair. Metabolites generated through microbial metabolism or shared microbial–host pathways may therefore contribute to immune reprogramming, although their effects are highly context dependent and cannot be regarded as uniformly beneficial or detrimental. This narrative review examines short-chain fatty acids, tryptophan-derived indoles, secondary bile acids, succinate, lactate, trimethylamine N-oxide, polyamines, and aromatic metabolites, with particular attention to source attribution, anatomical compartment, host co-metabolism, receptor signaling, and their potential influence on antimicrobial immunity. These metabolites may act through G-protein-coupled receptors, AhR, PXR, FXR, TGR5, VDR, HIF-1α, inflammasome pathways, mitochondrial signaling, and epigenetic mechanisms to modulate macrophages, neutrophils, dendritic cells, innate lymphoid cells, T lymphocytes, epithelial barriers, and endothelial responses. The direction and magnitude of these effects are likely to vary according to timing, concentration, anatomical compartment, immune-cell state, pathogen burden, and renal or hepatic clearance. Within this framework, attenuation of inflammatory signaling may help limit early collateral tissue injury, yet the same response could become disadvantageous when antimicrobial competence is already impaired. Similarly, succinate and lactate may reflect or contribute to distinct inflammatory, regulatory, or reparative programs depending on whether they arise from microbial or host metabolism and whether they act within intracellular or extracellular compartments. Human studies in critically ill populations have linked dysbiosis and metabolomic alterations with secondary infection, prolonged organ support, and mortality; however, most have not been able to resolve microbial source attribution or establish causal mediation. Future work would therefore benefit from integrating longitudinal microbiome, metabolome, immune-function, and organ-clearance measurements with source-resolved approaches, including isotope tracing, as well as state-specific and trajectory-informed clinical endpoints. Microbiome-directed interventions may be most appropriately investigated as context-dependent modulators of host defense rather than as broadly anti-inflammatory strategies.

## 1 Introduction

Critical illness can substantially disrupt the intestinal microbiota–metabolite–host immune axis while simultaneously giving rise to profound and rapidly evolving immune dysfunction. Infection, trauma, burns, acute respiratory distress syndrome (ARDS), shock, and major surgery can provoke intense innate immune activation, endothelial injury, coagulation disturbances, mitochondrial stress, and organ dysfunction. Importantly, however, critical illness does not correspond to a single, uniform immunological state. Even during the first days, anti-inflammatory circuits, lymphocyte apoptosis, monocyte deactivation, and inhibitory checkpoint signaling may develop alongside hyperinflammation. Longitudinal studies have therefore prompted a reassessment of the traditional model of a uniform transition from systemic inflammation to a delayed compensatory anti-inflammatory phase. Instead, patients may follow heterogeneous and overlapping immune trajectories, including rapid resolution, persistent hyperinflammation, predominant immunosuppression, or persistent inflammation–immunosuppression–catabolism associated with chronic critical illness.

Human evidence further supports this dynamic view of host immunity. Postmortem tissues from patients who died of sepsis showed profound loss and functional impairment of immune effector cells, increased inhibitory receptor expression, and diminished cytokine production despite ongoing infection and organ failure (Boomer et al., 2011). In prospective septic-shock cohorts, persistently low monocyte human leukocyte antigen-DR (mHLA-DR) was associated with nosocomial infection (Landelle et al., 2010), whereas patients who developed chronic critical illness exhibited sustained lymphopenia, low mHLA-DR, increased soluble PD-L1, and more secondary infections than those who recovered rapidly (Stortz et al., 2018). Circulating myeloid-derived suppressor cells may remain expanded for weeks, suppress T-cell proliferation, express ARG1, and correlate with nosocomial infection and poor functional outcomes (Mathias et al., 2017). Ex vivo blockade of PD-1 or PD-L1 partially restored lymphocyte survival and cytokine production in samples from patients with sepsis (Chang et al., 2014a, 2014b). Collectively, these findings support the presence of clinically relevant immune dysfunction while also highlighting an important interpretive challenge: a low-inflammatory phenotype may reflect adaptive restraint, impaired antimicrobial competence, terminal cellular failure, or a combination of these states.

The intestinal microbiota may influence these immune trajectories through concurrent changes in ecological structure, metabolic activity, and barrier function. Critical illness is often accompanied by a rapid decline in microbial diversity, expansion of opportunistic pathogens, and disruption of cross-feeding networks that normally generate short-chain fatty acids (SCFAs), indoles, secondary bile acids, and polyamines. At the same time, oxygen exposure, vasopressors, bowel hypoperfusion, fasting, interruptions of enteral nutrition, antimicrobial therapy, proton-pump inhibitors, opioids, sedatives, renal replacement therapy, and hepatic dysfunction can alter both microbial metabolic output and host metabolism and clearance. Loss of intestinal barrier integrity may further increase the access of microbial products and metabolites to systemic compartments. The biologically relevant exposure is therefore better understood not simply as the abundance of an individual taxon or the concentration of a single circulating metabolite, but as a dynamic flux shaped by microbial metabolic capacity, substrate availability, epithelial transport, host biotransformation, organ clearance, and tissue-specific receptor expression. This distinction is particularly important for metabolites such as succinate, lactate, polyamines, and several aromatic compounds that may arise from both microbial and host pathways.

This review examines how metabolites generated by microbial metabolism or combined microbial–host pathways may influence host immunity across different immune states and trajectories of critical illness. Rather than organizing the evidence around a single diagnosis or treating circulating metabolites as direct proxies of intestinal microbial activity, we adopt a state and context-dependent framework centered on four questions: where a metabolite originates, in which anatomical compartment it acts or is measured, in which immune state it is encountered, and whether its effects preserve or impair host defense. We focus on eight metabolite groups: SCFAs; indoles and related tryptophan metabolites; secondary bile acids; succinate; lactate; trimethylamine N-oxide (TMAO); polyamines; and aromatic acids, including indoxyl sulfate, p-cresyl sulfate, phenylacetic acid, and phenylacetylglutamine. We distinguish metabolites produced predominantly by microorganisms from those generated through combined microbial and host metabolism, and we separate human associations from causal preclinical evidence. Our central thesis is that metabolite effects are shaped by source, timing, dose, anatomical compartment, immune-cell state, pathogen burden, and organ function. This framework may help explain why the same molecule can reduce tissue injury in one setting yet impair antimicrobial defense in another, and why interventions based solely on increasing or suppressing a circulating metabolite may have limited applicability across heterogeneous ICU populations.

## 2 Narrative review approach and evidence framework

This narrative review was informed by targeted searches of PubMed and Europe PMC from database inception through 1 August 2026. Search terms included combinations related to critical illness, sepsis, acute respiratory distress syndrome (ARDS), trauma, immune reprogramming, immunosuppression, secondary infection, gut microbiota, microbiome, metabolomics, individual metabolite classes, host co-metabolism, and their cognate receptors. No formal language restriction was applied, although the final synthesis predominantly included English-language publications accessible to the authors. Scopus and Web of Science were considered as supplementary bibliographic resources but were not searched using a separate predefined systematic strategy. Forward and backward citation tracking was used to identify additional foundational and mechanistic studies.

Priority was given to original human cohort studies, longitudinal investigations, randomized trials, germ-free or gnotobiotic experiments, isotope-tracing studies, receptor-deficient models, and studies that directly examined microbial source attribution or microbial–host metabolic transformation. Review articles were used to provide background and identify relevant primary studies but were not treated as primary causal evidence. Studies reporting null, conflicting, or context-dependent findings were also incorporated when they contributed meaningfully to the interpretation of source attribution, biological directionality, or translational relevance. Because study identification and selection were iterative rather than based on a prospectively registered systematic search, a formal PRISMA-style screening process was not undertaken. The final narrative synthesis included approximately 90 cited publications, comprising original human and experimental studies together with selected reviews and contextual sources. This numerical estimate is intended to indicate the approximate scale of the evidence base rather than to suggest systematic-review completeness. As the objective was to develop a mechanistic and context-dependent synthesis rather than to estimate pooled effect sizes or perform formal evidence grading, the literature search and study selection remained iterative and consistent with a narrative-review design. The search should therefore be interpreted as a targeted rather than exhaustive appraisal of the literature, and it is possible that some eligible positive, null, negative, or discordant findings were not captured.

In interpreting the literature, we distinguished among longitudinal human evidence, cross-sectional human associations, causal animal or ex vivo experiments, and mechanistic evidence derived from experimental systems outside critical illness. Greater interpretive weight was given to longitudinal human studies and experimental designs that directly perturbed the metabolite, receptor, microbial producer, host metabolic pathway, or relevant microbial–host metabolic step. The strength of inference was considered lower when microbial origin was inferred solely from database annotation, when renal or hepatic clearance was not evaluated, when host production could not be excluded, or when a single plasma measurement was used as a surrogate for intestinal metabolite production. Comparative judgments regarding evidence strength were therefore based on qualitative consideration of study design, biological context, source attribution, longitudinal structure, and experimental perturbation rather than on a formal risk-of-bias assessment or certainty-of-evidence grading framework. Throughout this review, the term “microbiota-derived” is reserved for metabolites for which microbial participation in production or transformation is demonstrated or biologically well established. For metabolites with substantial host contribution, we explicitly distinguish microbial, host, and combined microbial–host sources rather than treating circulating concentration as a direct proxy of intestinal microbial activity.

## 3 Critical illness as a dynamic immune-metabolic ecosystem

### 3.1 Hyperinflammation and immunosuppression coexist

The early phase of critical illness is commonly characterized by activation of pattern-recognition receptors, complement and coagulation cascades, inflammasome signaling, emergency myelopoiesis, endothelial activation, and a shift toward aerobic glycolysis in innate immune cells. Metabolites are increasingly recognized not simply as passive readouts of these processes, but also as active participants in immune regulation. Intracellular succinate stabilizes HIF-1α and promotes IL-1β production in activated macrophages (Tannahill et al., 2013), whereas extracellular lactate can suppress Toll-like receptor and inflammasome signaling through GPR81 (Hoque et al., 2014). These apparently divergent effects illustrate how metabolite signaling may vary according to cellular source, anatomical compartment, receptor engagement, and immune-cell state, and may occur concurrently across different tissues or cell populations.

Immune suppression can also emerge early in the course of critical illness. In a longitudinal study of severe sepsis, monocyte antigen-presentation defects, T-cell apoptosis, impaired proliferative responses, and regulatory cytokines were already detectable during the initial phase and showed distinct relationships with mortality and secondary infection (Gomez et al., 2014). Recent prospective phenotyping across mild infection, non-infectious critical illness, and sepsis further suggests that some canonical “sepsis immunosuppression” markers may vary along a continuum of illness severity rather than define a discrete syndrome (Snow et al., 2025). A 20-year cohort of 1,023 patients with septic shock nevertheless supports persistent low mHLA-DR as a clinically relevant correlate of mortality and ICU-acquired infection (Monneret et al., 2025). Taken together, these findings suggest that immune markers may be most informative when interpreted longitudinally: an isolated value may reflect disease severity, adaptive regulation, or functional impairment, whereas persistence and failure of recovery may provide greater biological and clinical context.

Epigenetic remodeling may provide a mechanistic link between inflammatory activation, tolerance, and subsequent immune dysfunction. Repeated endotoxin exposure appears to induce gene-specific tolerance rather than global immune paralysis. Human monocytes fail to reacquire active chromatin marks at selected inflammatory, lipid-metabolic, and phagocytic loci, and β-glucan can partially reverse this state (Novakovic et al., 2016). IFN-γ can restore access of chromatin-remodeling machinery to tolerant cytokine promoters without necessarily correcting every proximal Toll-like receptor defect (Chen and Ivashkiv, 2010). Metabolites arising from microbial or host pathways may intersect with these processes through histone deacetylase inhibition, protein acylation, changes in redox cofactors, or altered mitochondrial substrate use. Such mechanisms provide a biologically plausible route through which metabolic exposures may reinforce or redirect immune-cell functional states, although their specific contribution to immune memory in human critical illness remains incompletely defined.

### 3.2 From rapid recovery to chronic critical illness

Three broad trajectories may provide a useful clinical framework. In rapid recovery, pathogen control and tissue repair become progressively coordinated, organ support can be withdrawn, and immune competence begins to recover. By contrast, early death is typically associated with persistent inflammation, shock, endothelial dysfunction, and metabolic failure. Chronic critical illness is characterized by a different pattern, in which low-grade inflammation may coexist with immunosuppression, recurrent infection, protein catabolism, weakness, and prolonged dependence on organ support. These trajectories are not necessarily fixed and may shift following secondary infection, invasive procedures, or other superimposed insults.

Human multiomic studies further illustrate this biological heterogeneity. An integrated clinico-metabolomic model improved mortality prediction in sepsis beyond clinical variables alone (Langley et al., 2013). Targeted and untargeted metabolomic studies have associated mortality with disturbances in lipids, amino acids, kynurenine, lactate, bile acids, and markers of mitochondrial stress (Ferrario et al., 2016; Kosyakovsky et al., 2022; Rogers et al., 2024). In mechanically ventilated patients with septic shock, baseline acylcarnitine and amino-acid profiles were associated with time to extubation, freedom from vasopressors, and progression toward chronic critical illness (Jennaro et al., 2022). Longitudinal multiomic analysis of ARDS and sepsis identified mortality-associated programs characterized by combinations of glycolysis and innate immune activation, hepatic and immune dysfunction with impaired fatty-acid oxidation, interferon suppression with altered mitochondrial respiration, and redox failure (Alipanah-Lechner et al., 2026).

Taken together, these studies suggest that metabolic and immune trajectories evolve in parallel during critical illness, although they do not by themselves establish that the observed circulating metabolites are microbiota-derived or that metabolic alterations directly drive immune dysfunction. Host tissue injury, nutrition, medications, renal and hepatic clearance, and microbial metabolism may all contribute to the measured signatures, while baseline host factors and time-varying ICU exposures can further modify these relationships. These considerations are important when interpreting clinical metabolomic data in the context of microbiota–host mechanisms. The major biological domains and their potential bidirectional interactions are summarized descriptively in Figure 1.

**Figure 1:**
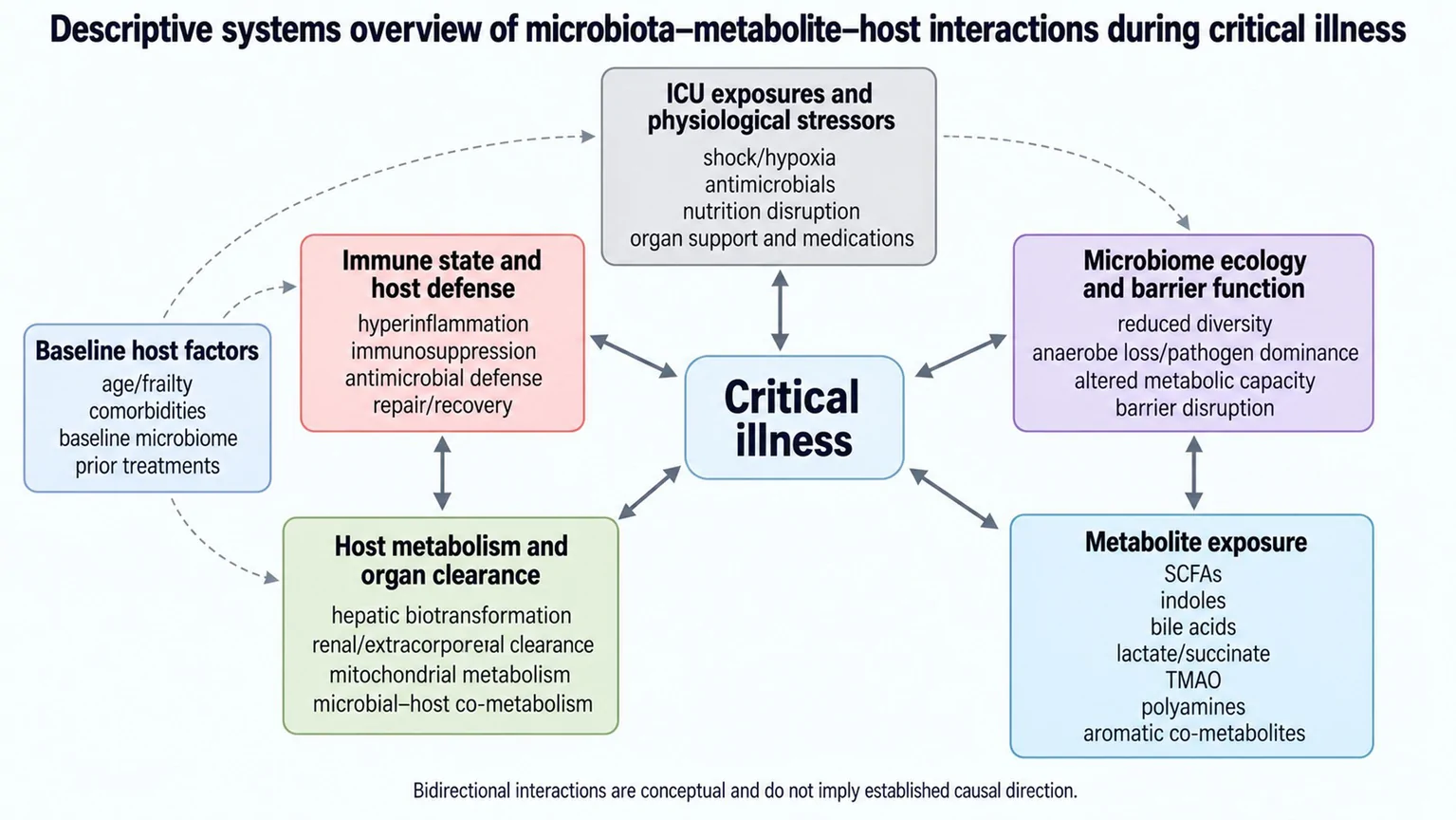
Descriptive systems overview of microbiota–metabolite–host interactions during critical illness. Critical illness alters intestinal microbial ecology, epithelial barrier integrity, nutritional substrate availability, host metabolism, organ clearance, and immune function through interacting physiological and treatment-related exposures. Baseline host factors further modify these relationships. Microbial and microbial–host metabolites are embedded within a bidirectional network rather than a unidirectional causal chain. Arrows indicate potential reciprocal interactions and do not imply established causal direction. The figure is intended as a descriptive synthesis of the biological domains discussed in this review.

## 4 Why metabolite exposure changes in the ICU

### 4.1 Ecological collapse and loss of metabolic redundancy

The healthy gut community contains overlapping fermentative and cross-feeding networks that provide functional redundancy in nutrient utilization and metabolite production. During critical illness, however, this ecological organization may become markedly disrupted, and prolonged ICU stays can be associated with ultra-low-diversity communities dominated by a single pathogen (Zaborin et al., 2014; McDonald et al., 2016). Pathogen colonization at ICU admission has been associated with subsequent infection or death (Freedberg et al., 2018), and prospective studies in mechanically ventilated patients have linked gut dysbiosis with mortality (Lamarche et al., 2018; Zhou et al., 2023). At the same time, disease severity, antimicrobial exposure, nutrition, and other ICU interventions change dynamically, which can complicate interpretation of the directionality and causal basis of these associations (Ojima et al., 2022).

Ecological disruption may alter metabolic function even before pronounced taxonomic changes become apparent. Loss of obligate anaerobes may reduce fermentation and secondary bile-acid conversion; depletion of cross-feeding organisms may favor accumulation of intermediates such as succinate; and expansion of opportunistic pathogens may introduce or amplify alternative metabolic outputs, including lactate and aromatic products. Anti-anaerobic antibiotic exposure has been associated with worse outcomes in critically ill patients, a finding that is consistent with, but does not on its own establish, a contribution of impaired anaerobic microbial function to adverse host responses (Chanderraj et al., 2023). Longitudinal multi-compartment profiling in acute respiratory failure further suggests that host–microbiota relationships are dynamic and may vary across both time and anatomical sites (Kitsios et al., 2024). Collectively, these observations are compatible with a functional view of dysbiosis in which loss of metabolic redundancy may be as biologically relevant as changes in taxonomic composition.

### 4.2 Barrier failure, compartment, and source attribution

Shock, hypoxia, epithelial apoptosis, impaired motility, and altered luminal nutrition can compromise intestinal barrier integrity and reshape the local metabolic environment. Reduced luminal butyrate may impair epithelial oxygen consumption and HIF-dependent barrier protection, whereas SCFAs can reinforce epithelial HIF signaling and tissue barrier function in experimental systems (Kelly et al., 2015). As intestinal permeability increases, the biological effect of a metabolite is likely to depend not only on its concentration but also on the compartment in which it is produced, transported, measured, or retained. Millimolar luminal butyrate, micromolar portal exposure, and substantially lower peripheral concentrations therefore represent biologically distinct exposure states and are unlikely to be directly interchangeable in experimental interpretation.

Source attribution is particularly challenging for metabolites such as succinate and lactate, which can be generated in substantial amounts by both host cells and microorganisms. TMAO, phenylacetylglutamine, indoxyl sulfate, and p-cresyl sulfate may be more appropriately regarded as meta-organismal products, in which microbial precursor generation is followed by host biotransformation. Their circulating concentrations are further influenced by renal excretion, hepatic metabolism, protein binding, and extracorporeal clearance. Bile-acid profiles are similarly shaped by hepatic synthesis and conjugation, enterohepatic circulation, microbial deconjugation and transformation, cholestasis, medications, and nutritional substrate availability. Consequently, an observed association between a circulating metabolite and a clinical outcome may reflect microbial activity, altered host metabolism, impaired clearance, tissue release, or some combination of these processes rather than a direct microbiota-derived signal.

More informative source-attribution studies therefore tend to integrate complementary approaches, including metagenomic pathway analysis, paired fecal and plasma metabolomics, microbial culture, isotope-labeled substrates, gnotobiotic or antibiotic-perturbation models, and explicit assessment of organ function. A representative example is the study of gut-derived succinate in intestinal ischemia–reperfusion, in which microbiome profiling, germ-free and antibiotic-treated animals, tissue metabolite measurements, SUCNR1 perturbation, and a human cardiopulmonary-bypass cohort were integrated to link microbial succinate production with lung injury (Wang et al., 2023). Comparable source-resolved evidentiary chains remain relatively uncommon in sepsis and other forms of critical illness. This limitation is therefore important to bear in mind when circulating metabolites are interpreted as direct readouts of intestinal microbial activity.

## 5 Major metabolite classes and state-dependent immune effects

### 5.1 Short-chain fatty acids: immune restraint, barrier support, and a host-defense trade-off

Acetate, propionate, and butyrate are produced predominantly through microbial fermentation of dietary substrates. They signal through FFAR2/GPR43, FFAR3/GPR41, and HCAR2/GPR109A, while butyrate and propionate can also inhibit histone deacetylases (HDACs), serve as carbon sources, and contribute to protein acylation. Through these mechanisms, microbial fermentation may influence epithelial integrity, innate immune regulation, and adaptive immune differentiation.

Foundational studies showed that butyrate promotes colonic regulatory T-cell differentiation through enhanced histone acetylation at the Foxp3 locus (Furusawa et al., 2013), whereas butyrate and propionate can support peripheral Treg generation (Arpaia et al., 2013). GPR109A signaling in colonic macrophages and dendritic cells promotes IL-10-producing and Treg-supportive programs and restrains inflammation (Singh et al., 2014). In intestinal macrophages, butyrate suppresses LPS-induced IL-6, IL-12, and nitric oxide through HDAC inhibition, providing a mechanism for commensal tolerance (Chang et al., 2014a, 2014b). In an endotoxemia model, butyrate increased IL-10 and attenuated inflammatory injury (Wang et al., 2017). Taken together, these studies are consistent with barrier-protective and immunoregulatory effects of SCFAs, although much of the mechanistic evidence derives from intestinal homeostasis or experimental inflammatory models rather than from human critical illness.

Importantly, immune restraint does not necessarily imply enhanced antimicrobial defense. Butyrate altered neutrophil maturation, reduced NADPH oxidase components and secondary granule enzymes, and impaired early control of *Pseudomonas aeruginosa* in the lung despite increased neutrophil recruitment (Dang et al., 2023). In contrast, butyrate enhanced macrophage ROS-dependent killing and survival in experimental leptospirosis through an MCT–HDAC3 axis (Chen et al., 2024). These divergent findings suggest that the consequences of SCFA exposure may vary according to pathogen identity, immune-cell type, exposure duration, tissue compartment, and the antimicrobial mechanism involved. Reduced inflammatory signaling therefore needs to be interpreted cautiously and does not, on its own, imply improved host defense.

In critical illness, low fecal SCFA concentrations may reflect depletion of anaerobic fermenters, reduced fiber delivery, altered intestinal transit, or increased epithelial utilization rather than a single microbial defect. Experimental restoration of SCFAs may support barrier function in selected settings, particularly during recovery, although systemic or high-dose exposure during active infection could potentially suppress effector functions required for pathogen clearance. Against this background, a general strategy of simply “restoring SCFAs” may be too broad to serve as a sufficiently specific therapeutic hypothesis. Future studies would benefit from specifying the individual SCFA, route and compartment of delivery, timing, microbial context, and immune state, while evaluating antimicrobial competence and pathogen clearance alongside inflammatory and barrier-related endpoints.

### 5.2 Tryptophan metabolism: microbial indoles, host kynurenine, and receptor-selective signaling

Tryptophan lies at the intersection of microbial and host metabolic pathways. Host indoleamine 2,3-dioxygenase generates kynurenine; enterochromaffin and other host pathways contribute serotonin-related products; and gut microorganisms generate indole, indole-3-aldehyde, indole-3-propionic acid (IPA), indole-3-acetic acid (IAA), indole lactic acid, and related derivatives. Many microbial indoles signal through the aryl hydrocarbon receptor (AhR) or pregnane X receptor (PXR), although their effects can vary substantially according to ligand identity, affinity, stability, concentration, anatomical compartment, and cell-specific receptor context.

Microbial indole-3-aldehyde activates AhR in innate lymphoid cells and supports IL-22-dependent mucosal protection (Zelante et al., 2013). Indole metabolites can also engage intestinal PXR to restrain TLR4 signaling and maintain barrier integrity (Venkatesh et al., 2014). Together, these mechanisms provide a biologically plausible connection between microbial tryptophan metabolism and protection against barrier dysfunction or microbial translocation. In experimental sepsis, IPA supplementation improved gut microbial disturbance and tissue injury (Fang et al., 2022), whereas another study linked microbiota-derived IPA to partial AhR activation, enhanced macrophage phagocytosis, and attenuated septic injury (Huang et al., 2022). IAA protected against sepsis-associated encephalopathy through microglial AhR signaling (Huang et al., 2025a, 2025b), and recent preclinical work attributed protection from sepsis-associated acute lung injury to Blautia-derived IAA and CD8 T-cell regulation (Chao et al., 2026). Limosilactobacillus reuteri-derived indole-3-carboxaldehyde enhanced macrophage bactericidal activity through DUSP1/ERK/NOX2/ROS signaling (Li et al., 2026). Collectively, these studies are consistent with metabolite–receptor–immune interactions, although most of the evidence remains preclinical and has not yet established reproducible exposure–response relationships in critically ill patients.

The indole family is therefore better viewed as a collection of biologically distinct exposures rather than as a single uniform pathway. Oxindole suppressed CXCL13 and reduced acute lung injury in one recent model (Tang et al., 2026), illustrating potentially beneficial anti-inflammatory signaling, whereas AhR activation in other contexts can also support regulatory programs that may be less favorable during persistent infection. Moreover, reduced microbial indole production and increased host kynurenine flux may occur simultaneously. In sepsis, a higher kynurenine-to-tryptophan ratio was associated with immune and microvascular dysfunction (Darcy et al., 2011); longitudinal septic-shock data demonstrated sustained disruption of tryptophan catabolites and related enzymes (Troché et al., 2020); and greater kynurenine-pathway activity was associated with hospital mortality in ARDS (Chiu et al., 2022). Kynurenine is primarily a host inflammatory metabolite and is therefore more appropriately interpreted as a marker of host tryptophan metabolism than as evidence of increased microbial tryptophan metabolism. Analyses that combine microbial indoles and host kynurenine products within a single “microbiota pathway” may therefore obscure biologically distinct processes.

A more informative framework would separate microbial indole availability from host kynurenine activation and evaluate both in relation to immune state and host-defense status. During barrier injury, selected microbial AhR/PXR ligands may warrant investigation for their effects on epithelial integrity and antimicrobial defense. During established immunosuppression, the more relevant question may be whether bactericidal indole signaling can be preserved without further reinforcing host kynurenine-associated T-cell dysfunction. Total tryptophan concentration or a single kynurenine-to-tryptophan ratio is unlikely to fully distinguish these separate microbial and host metabolic states.

### 5.3 Secondary bile acids: receptor context, concentration, and inflammasome direction

Primary bile acids synthesized by the liver are modified by intestinal microorganisms through deconjugation, dehydroxylation, epimerization, and oxidation, generating a chemically diverse pool of secondary bile acids. These metabolites can signal through nuclear receptors such as FXR and VDR as well as the membrane receptor TGR5, while some bile-acid species may also influence membrane integrity and mitochondrial stress. In critical illness, the measured bile-acid profile is shaped not only by microbial transformation but also by fasting, enteral nutrition, cholestasis, hepatic dysfunction, antimicrobial exposure, and loss of bile-acid-transforming organisms.

Specific secondary bile-acid metabolites can influence adaptive immune differentiation. 3-oxo-lithocholic acid inhibits Th17 differentiation by binding RORγt, whereas isoallo-lithocholic acid promotes Treg differentiation through mitochondrial reactive oxygen species (Hang et al., 2019). Bile-acid activation of TGR5 can increase cAMP signaling and suppress NLRP3 inflammasome activation (Guo et al., 2016). Together, these findings support a mechanistic link between microbial bile-acid transformation, inflammatory signaling, and T-cell differentiation, although the relevance of these pathways to immune trajectories in human critical illness remains incompletely established.

The biological effects of bile-acid signaling are not uniform across different contexts. Chenodeoxycholic acid activated NLRP3 through reactive oxygen species and potassium efflux and contributed to cholestatic fibrosis in mice (Gong et al., 2016). In severe acute pancreatitis, changes in conjugated bile acids were linked to TGR5/NLRP3 signaling and inflammatory injury (Zhang et al., 2024). Human sepsis studies have identified prognostic abnormalities in secondary and conjugated bile-acid species, although such associations do not by themselves clarify whether the altered profiles primarily reflect microbial transformation, hepatic dysfunction, cholestasis, or combinations of these processes (Xu et al., 2026). Given the substantial variation in receptor affinity, conjugation state, tissue exposure, and detergent properties among individual bile acids, the term “bile acids” may be too broad to represent a single biological or therapeutic class. Interpretation is therefore likely to be most informative when molecular speciation, conjugation status, anatomical compartment, timing, liver function, and, where feasible, microbial bile-acid transformation capacity are considered together.

### 5.4 Succinate: intracellular inflammatory signal, extracellular receptor ligand, and microbial cross-feeding intermediate

Succinate provides a useful example of why source and anatomical compartment are important for interpretation. Intracellular succinate accumulates in activated macrophages when the tricarboxylic-acid cycle is remodeled; it inhibits prolyl hydroxylases, stabilizes HIF-1α, and promotes IL-1β production (Tannahill et al., 2013). Ischemic tissues and microorganisms can also release succinate into extracellular compartments. Extracellular succinate is sensed through SUCNR1/GPR91 on macrophages, dendritic cells, tuft cells, and other cell types, although the resulting phenotype may vary according to receptor expression, tissue context, and the cellular state in which signaling occurs.

In the small intestine, microbial or protist-derived succinate activates SUCNR1 on tuft cells and initiates an IL-25–ILC2 type-2 immune circuit (Nadjsombati et al., 2018). Such signaling can promote mucus production and epithelial defense. In macrophages, however, SUCNR1-associated effects appear to be strongly context dependent. Gut microbiota-derived succinate aggravated intestinal ischemia–reperfusion-associated lung injury through SUCNR1, PI3K–AKT, HIF-1α, alveolar macrophage polarization, and epithelial apoptosis (Wang et al., 2023). By contrast, myeloid SUCNR1 supported an anti-inflammatory macrophage program and responses to type-2 cytokines in metabolic inflammation (Keiran et al., 2019). Taken together, these findings suggest that intracellular succinate accumulation and extracellular SUCNR1 activation represent related but biologically distinct forms of exposure and are unlikely to be directly interchangeable in biological interpretation.

Critical illness may increase host-derived succinate through hypoxia, mitochondrial dysfunction, ischemia–reperfusion, and leukocyte activation while simultaneously increasing luminal microbial succinate through disruption of succinate-consuming cross-feeding networks. Plasma or tissue succinate concentrations alone are unlikely to distinguish reliably among these potential sources. Consequently, elevated succinate may reflect tissue hypoxia, immune-cell metabolic remodeling, microbial production, altered clearance, or some combination of these processes. Whether attenuation of downstream succinate signaling would be beneficial is therefore likely to vary according to source, compartment, immune state, and receptor context. Future studies would benefit from combining isotope tracing or source-resolved metagenomics with compartment-specific measurements and from distinguishing macrophage-intrinsic HIF-1α signaling from extracellular SUCNR1 biology before succinate-directed interventions are evaluated.

### 5.5 Lactate and lactylation: danger signal, negative feedback, and persistence niche

Lactate is better considered as a biologically heterogeneous exposure rather than as a single uniform signal. In critical illness, circulating lactate predominantly reflects host metabolic processes, including tissue hypoperfusion, adrenergic stimulation, impaired clearance, and aerobic glycolysis in activated immune cells, whereas microbial fermentation can contribute lactate within intestinal or infection-associated compartments. Where analytically relevant, L and D-lactate are best considered separately because their sources, relative abundance, and biological interpretation may differ. Lactate functions both as a metabolic substrate and as a signaling molecule. Extracellular lactate can act through HCAR1/GPR81 and monocarboxylate transporters, alter the NADH/NAD + balance, and influence transcriptional programs. Intracellular lactate can also contribute lactyl groups to histone and non-histone proteins, thereby linking glycolytic flux to epigenetic and post-translational regulation.

Histone lactylation was initially described as a delayed program in glycolytic macrophages associated with expression of genes involved in homeostasis and wound repair (Zhang et al., 2019). This finding provides a mechanistic framework in which sustained glycolytic metabolism may contribute to later reparative programs, although the functional consequences of lactylation are likely to vary according to cell type, timing, and tissue context. Extracellular lactate can also suppress innate inflammatory signaling: GPR81 activation reduced Toll-like receptor and inflammasome-mediated liver and pancreatic injury (Hoque et al., 2014), and lactate inhibited macrophage responses to LPS through GPR81, YAP, and NF-κB (Yang et al., 2020). Such effects may help limit collateral tissue injury during hyperinflammatory states, although they do not necessarily imply preservation of antimicrobial competence.

In some settings, however, the same negative-feedback mechanisms may become maladaptive. In a sepsis model, macrophage metabolic signaling through the Spns2/S1P axis linked lactate-associated ROS to early hyperinflammation and later immunosuppression; correction of this axis improved outcomes across both states (Fang et al., 2023). *Enterococcus faecalis*-derived lactic acid suppressed macrophage activation through MCT1/GPR81 and facilitated persistent polymicrobial wound infection (da Silva et al., 2026). GPR81 activation also delayed inflammatory resolution in experimental acute lung injury (Liang et al., 2026). Taken together, these observations suggest that reduced inflammatory output is not necessarily equivalent to effective resolution: suppression of macrophage activation may reduce tissue injury, but under conditions of impaired pathogen clearance it may also favor a permissive environment for microbial persistence.

Clinical lactate is an established severity marker but does not specifically reflect microbial lactate production or immune-cell lactylation. Translational studies would therefore benefit from distinguishing L and D-lactate where relevant, while also considering tissue versus plasma concentrations, cellular lactylation, microbial lactate-production capacity, organ clearance, and concurrent catecholamine exposure. Evaluation of bacterial clearance and secondary infection may also provide a more complete assessment than reliance on cytokine endpoints alone. Experimental stimulation of pyruvate dehydrogenase complex activity improved immunometabolic homeostasis, bacterial clearance, and survival in septic mice (McCall et al., 2018), suggesting that restoration of mitochondrial pyruvate oxidation may offer a mechanistically distinct alternative to indiscriminate lactate antagonism.

### 5.6 TMAO: a meta-organismal inflammatory and thrombotic signal with a nutritional paradox

Gut microorganisms convert dietary choline and carnitine to trimethylamine, which is subsequently oxidized by hepatic flavin-containing monooxygenases to trimethylamine N-oxide (TMAO). TMAO is therefore more appropriately regarded as a meta-organismal metabolite rather than a purely microbial product. Mechanistic evidence from vascular and platelet biology suggests that TMAO can increase platelet responsiveness and thrombosis potential through intracellular calcium signaling (Zhu et al., 2016); colonization with CutC-containing microbial communities can transfer this phenotype (Skye et al., 2018); and non-lethal inhibition of microbial CutC/D reduces TMAO production and thrombosis in animal models (Roberts et al., 2018). TMAO has also been reported to activate endothelial NLRP3 through SIRT3–SOD2–mitochondrial ROS signaling (Chen et al., 2017) and to promote CCR2-positive monocyte/macrophage infiltration after kidney ischemia–reperfusion (Ren et al., 2024).

These mechanisms may be relevant to endothelial injury, immunothrombosis, and acute kidney injury in critical illness, although the available sepsis data are more complex. In 95 mechanically ventilated patients with sepsis, lower rather than higher plasma TMAO was associated with poorer nutritional status and longer prior antibiotic exposure, and septic patients had lower TMAO concentrations than non-septic cardiovascular controls (Chou et al., 2021). Circulating TMAO is therefore likely to reflect not only microbial trimethylamine production but also dietary substrate availability, hepatic oxidation, antimicrobial exposure, renal clearance, and catabolic state.

Against this background, cardiovascular associations between higher TMAO and adverse outcomes may not be directly generalizable to heterogeneous ICU populations. Low TMAO may reflect reduced substrate intake, antibiotic-associated loss of microbial metabolic capacity, or malnutrition, whereas elevated TMAO during renal dysfunction may partly reflect impaired excretion rather than increased microbial production. The same circulating concentration may therefore arise from biologically distinct production–clearance states. TMAO may consequently be more informative when interpreted as a context-sensitive meta-organismal exposure rather than as an isolated marker of gut microbial activity or a stand-alone sepsis biomarker.

### 5.7 Polyamines: repair, efferocytosis, and immune suppression on the same axis

Putrescine, spermidine, and spermine arise from both host and microbial arginine/ornithine metabolism, making attribution to a single source inherently difficult. These polyamines contribute to nucleic-acid stability, translation, mitochondrial function, autophagy, and epithelial proliferation. Spermidine is also required for hypusination of eIF5A, a modification involved in the regulation of translation and immune-cell metabolism. Because host tissues and immune cells can both synthesize and import polyamines, fecal or plasma concentrations are unlikely to provide a direct measure of microbial production.

Polyamines may also contribute to restraint of excessive innate immune activation. Spermine uptake suppresses monocyte pro-inflammatory cytokine synthesis (Zhang et al., 1999), and delayed spermine treatment reduced inflammatory mediators and improved survival in experimental polymicrobial sepsis (Zhu et al., 2009). After efferocytosis, macrophages increase polyamine import, and blocking this process prevents suppression of IL-1β and IL-6, consistent with a role for polyamines in pro-resolving immune reprogramming (McCubbrey et al., 2022). Microbial polyamine biosynthesis can also support epithelial renewal and anti-inflammatory colonic macrophage differentiation, as shown using wild-type and polyamine-deficient *Escherichia coli* in germ-free mice (Nakamura et al., 2021). Collectively, these findings suggest that the biological effects of polyamines are shaped both by cellular uptake and by the context in which exposure occurs.

Adaptive immune effects appear to be similarly context dependent. T cells use both *de novo* synthesis and extracellular salvage to maintain their polyamine pool, and combined blockade impairs proliferation and inflammatory function (Wu et al., 2020). Spermidine preserves autophagy and function in aged human CD8 T cells through eIF5A and TFEB (Alsaleh et al., 2020), and it can shift CD4 T-cell differentiation toward a regulatory phenotype (Carriche et al., 2021). Conversely, spermidine can suppress dendritic-cell activation and subsequent interactions with CD4 T cells through eIF5A hypusination and metabolic adaptation (Meehan et al., 2025). Thus, polyamine signaling may support epithelial repair, efferocytosis, and selected adaptive functions in some settings while restraining antigen presentation or inflammatory activation in others. Their biological significance is therefore likely to reflect not only extracellular concentration, but also source, cell-specific synthesis and uptake, immune state, and downstream hypusination-dependent programs.

### 5.8 Aromatic microbial–host co-metabolites: receptor signaling, mitochondrial stress, and organ-clearance dependence

Microbial metabolism of phenylalanine, tyrosine, and tryptophan generates aromatic precursors that undergo further host transformation into compounds such as phenylacetylglutamine (PAGln), p-cresyl sulfate, and indoxyl sulfate. These molecules may therefore be more appropriately viewed as microbial–host co-metabolites rather than exclusively microbial products. They may be particularly relevant in critical illness because acute kidney injury, hypoalbuminemia, and renal replacement therapy can alter their circulating concentrations, protein binding, and tissue exposure. Depending on the specific molecule and cellular context, aromatic co-metabolites may influence inflammatory signaling, immune restraint, endothelial function, platelet activation, or mitochondrial stress.

p-Cresyl sulfate suppressed LPS-induced IL-12, nitric oxide, and CD40 while increasing IL-10 in murine macrophages, a pattern compatible with reduced pro-inflammatory activation (Shiba et al., 2016). By contrast, indoxyl sulfate enhanced macrophage ROS, NF-κB, nitric oxide, TNF, and IL-6 responses to LPS (Adesso et al., 2013). Another study found that indoxyl sulfate induced pro-IL-1β through AhR–NF-κB/MAPK signaling without activating NLRP3 or efficiently generating mature IL-1β (Wakamatsu et al., 2018). Taken together, these findings suggest that aromatic co-metabolites can engage distinct inflammatory programs rather than producing a uniform immune phenotype.

PAGln provides a useful example of the meta-organismal interface between microbial aromatic-amino-acid metabolism and host adrenergic signaling. Microbial pathways generate phenylacetic acid, which is subsequently converted by the host to PAGln (Zhu et al., 2023). PAGln increases platelet responsiveness through adrenergic receptors (Nemet et al., 2020) and acts as an endogenous allosteric modulator of β2-adrenergic receptors (Saha et al., 2024). Phenylacetic acid has been linked experimentally to mitochondrial ROS generation and endothelial senescence (Saeedi Saravi et al., 2025), whereas PAGln has been implicated in a β2-adrenergic trained-inflammatory program in myeloid cells associated with impaired wound repair (Huang et al., 2025a, 2025b). These mechanisms may be relevant in catecholamine-treated critically ill patients, although direct evidence establishing their contribution to sepsis or ICU immune dysfunction remains limited.

Small clinical studies have associated phenyllactic and hydroxyphenyl acids with septic shock, lactate, catecholamine metabolites, and cardiovascular stress (Beloborodova et al., 2018). Causal interpretation remains challenging because microbial production, dietary substrate, organ dysfunction, vasopressor exposure, albumin concentration, and extracorporeal clearance are tightly coupled in critical illness. Future studies would benefit from assessing free and total metabolite fractions, renal function and dialysis timing, albumin, vasopressor exposure, and, where possible, microbial source pathways. In the absence of source-attribution evidence, circulating aromatic metabolites are therefore best interpreted cautiously rather than as direct readouts of intestinal microbial activity.

## 6 A unifying model: source, state, exposure, compartment, and host-defense competence

The biological meaning of a metabolite signal during critical illness is unlikely to be captured by concentration alone. Across the metabolite classes reviewed above, interpretation repeatedly appears to depend on five interacting dimensions: source, immune state, exposure, anatomical compartment, and host-defense competence.

First, *source* shapes what a measured metabolite is likely to represent. Some metabolites are produced predominantly by intestinal microorganisms, whereas others arise from host metabolism or microbial–host co-metabolism. For succinate, lactate, polyamines, TMAO, and several aromatic co-metabolites, interpretation of circulating concentrations is therefore likely to be more informative when accompanied by explicit consideration of source attribution. This distinction is particularly relevant because the same circulating analyte may reflect microbial production, host tissue release, impaired clearance, or some combination of these processes.

Second, *immune state and temporal context* modify the biological significance of the same pathway. Hyperinflammation, immune suppression, metabolic dysfunction, and tissue repair may coexist rather than occur as fixed sequential stages. A pathway that limits collateral inflammatory injury may be beneficial in one setting yet compromise antimicrobial competence when phagocytic, antigen-presenting, or lymphocyte function is already impaired. Metabolite-associated immune restraint is therefore best interpreted in the context of pathogen burden and functional host defense rather than classified as intrinsically beneficial or harmful.

Third, *dose, duration, and anatomical compartment* shape biologically effective exposure. Luminal, portal, systemic, and intracellular metabolite pools represent distinct biological contexts. Local mucosal signaling may occur without substantial systemic exposure, whereas renal or hepatic dysfunction may markedly alter circulating concentrations without a corresponding increase in microbial production. Protein binding, hepatic transformation, and extracorporeal clearance can further modify tissue exposure. Metabolite–response relationships are therefore likely to be more appropriately evaluated as context and exposure-dependent rather than assumed to follow a simple linear pattern.

Fourth, *cellular and tissue context* influences the direction of immune effects. The metabolite classes reviewed in Section 5 can affect epithelial barrier function, macrophage and neutrophil antimicrobial activity, antigen presentation, lymphocyte differentiation, endothelial activation, platelet function, and tissue repair through distinct mechanisms. Their biological effects may therefore be more meaningfully interpreted through specific functional outputs than through broad “pro-inflammatory” or “anti-inflammatory” labels. Reduced cytokine production accompanied by preserved or enhanced microbial killing may be compatible with immune resolution, whereas concurrent loss of inflammatory responsiveness and antimicrobial function may be more consistent with immune dysfunction.

Fifth, *organ function and treatment exposure are integral components of the biological system rather than merely nuisance covariates*. Renal and hepatic dysfunction can alter metabolite concentration, chemical form, protein binding, and clearance, while antibiotics, nutrition, vasopressors, and organ-support interventions can simultaneously modify microbial ecology, substrate availability, host metabolism, and immune function. Depending on timing and the estimand of interest, the same clinical variable may function as a confounder, mediator, effect modifier, or consequence of prior exposure. This distinction is important because broad or indiscriminate statistical adjustment may, in some settings, obscure rather than clarify causal relationships.

Taken together, these dimensions form the basis of a *source–state–exposure–competence framework*. Source describes whether a metabolite is predominantly microbial, host derived, or generated through microbial–host co-metabolism; *state* reflects the prevailing immune context; exposure incorporates concentration, duration, and anatomical compartment; and competence refers to whether the observed immune response remains compatible with effective antimicrobial defense. This framework supports the hypothesis that patients sharing the same clinical diagnosis may nevertheless exhibit biologically distinct microbiota–metabolite–immune relationships. It also provides a conceptual basis for testing whether source-resolved, longitudinal, and immune-function–enriched study designs offer greater biological interpretability than single circulating metabolite measurements alone.

Table 1 summarizes the major metabolite classes, their potential implications for host defense, and the principal experimental contexts supporting these relationships. Figure 1 provides a descriptive overview of the bidirectional interactions among baseline host factors, critical-illness exposures, microbial ecology, metabolite exposure, host metabolism and organ clearance, and immune state. Figure 2 complements this systems-level view with a hypothesis-generating causal framework for longitudinal analyses, in which microbial metabolic capacity, systemic metabolite exposure, immune dysfunction, treatment exposures, organ function, and clinical outcomes are positioned according to timing and the estimand of interest.

**Table 1:** Source-, context-, and evidence-dependent immune actions of major microbial and microbial–host co-metabolites.

Metabolite class	Principal microbial/host source	Major receptors or pathways	Context-dependent immune actions	Host-defense implications	Key interpretive caution	Evidence level/experimental context
Acetate, propionate, butyrate	Predominantly microbial fermentation; host uptake and oxidation	FFAR2, FFAR3, HCAR2, HDAC inhibition, epithelial HIF	Barrier support, Treg differentiation, and restraint of inflammatory signaling	May limit collateral injury, but effects on antimicrobial killing vary by pathogen, cell type, and exposure	Effects differ by molecule, dose, duration, pathogen, and luminal versus systemic exposure	Predominantly preclinical mechanistic evidence; limited human critical-illness evidence
Microbial indoles and related tryptophan metabolites	Microbial tryptophan metabolism generating indole and related derivatives	AhR, PXR, IL-22, DUSP1/ERK/NOX2	Barrier protection, IL-22-associated mucosal responses, and context-dependent macrophage phagocytic or bactericidal effects	Selected indoles may support epithelial integrity and antimicrobial defense, but effects vary by ligand, tissue, immune state, and pathogen context	Individual indoles should not be treated as a single exposure, and circulating concentrations do not necessarily establish intestinal microbial production	Predominantly preclinical mechanistic evidence; limited direct human critical-illness evidence
Host kynurenine/IDO pathway	Predominantly host tryptophan catabolism through IDO/TDO pathways	IDO activity and downstream kynurenine-pathway signaling	Immune regulation, altered T-cell function, and associations with microvascular or systemic immune dysfunction	Increased pathway activity may accompany impaired immune competence and adverse clinical outcomes in sepsis or ARDS	Kynurenine is primarily host derived and should not be interpreted as evidence of increased microbial tryptophan metabolism	Human critical-illness observational evidence plus mechanistic evidence
Bile-acid species and microbial bile-acid transformation	Hepatic synthesis and conjugation of primary bile acids followed by microbial deconjugation and secondary transformation	FXR, TGR5, VDR, RORγt, NLRP3	Species-specific modulation of inflammasome signaling and Th17/Treg differentiation	Effects may range from inflammatory restraint to tissue injury depending on molecular species, conjugation state, concentration, and tissue context	Primary, conjugated, and secondary bile acids should not be treated as a single exposure; interpretation requires molecular speciation, liver function, cholestasis, and microbial transformation capacity	Human critical-illness observational evidence plus predominantly preclinical mechanistic evidence
Succinate	Host TCA-cycle remodeling, ischemic tissues, and microbial fermentation	HIF-1α, SUCNR1, PI3K–AKT, tuft-cell IL-25 signaling	Intracellular inflammatory signaling or extracellular SUCNR1-dependent immune regulation	May amplify inflammatory injury or support type-2/repair-associated programs depending on source and compartment	Intracellular versus extracellular pools and host versus microbial sources are biologically non-equivalent	Predominantly animal and mechanistic evidence; limited source-resolved human critical-illness evidence
Lactate	Predominantly host glycolysis and tissue production in critical illness, with additional microbial lactate production in selected intestinal or infectious contexts	HCAR1/GPR81, MCTs, histone lactylation, redox pathways	Inflammatory negative feedback, metabolic signaling, and lactylation-associated transcriptional programs	May limit tissue injury but can also suppress macrophage antimicrobial activity and favor microbial persistence	Clinical lactate does not establish microbial production or immune-cell lactylation; L and D-lactate should be distinguished where relevant	Extensive human critical-illness evidence for circulating lactate as a clinical marker; immune-signaling and lactylation mechanisms predominantly preclinical
TMAO	Microbial TMA production followed by hepatic FMO oxidation	Platelet calcium signaling, NLRP3, SIRT3–SOD2–mtROS, CCR2	Platelet activation, endothelial inflammatory signaling, and monocyte/macrophage recruitment	Potential relevance to immunothrombosis and vascular injury, but direction in sepsis is not established	Diet, antibiotics, hepatic conversion, renal clearance, and nutritional state strongly determine concentration	Limited human critical-illness observational evidence; broader human non-critical-illness and preclinical mechanistic evidence
Polyamines	Shared microbial and host arginine/ornithine metabolism	eIF5A hypusination, TFEB, autophagy, mitochondrial pathways	Efferocytosis, epithelial repair, T-cell regulation, and restraint of innate activation	May support repair and selected adaptive functions but can also suppress antigen-presenting-cell activity	Cell-specific synthesis, uptake, salvage, and hypusination prevent simple source attribution	Predominantly preclinical and human non-critical-illness mechanistic evidence; limited direct human ICU evidence
Aromatic microbial–host co-metabolites	Microbial aromatic-amino-acid metabolism followed by host transformation/conjugation	AhR, NF-κB/MAPK, adrenergic receptors, mitochondrial ROS	Inflammatory signaling, immune restraint, platelet activation, endothelial and mitochondrial stress	Effects vary substantially among individual compounds and may influence vascular or immune dysfunction	Protein binding, kidney function, dialysis, albumin, vasopressors, and microbial source pathways must be considered	Limited human critical-illness observational evidence plus predominantly preclinical mechanistic evidence

Evidence-level descriptors indicate the principal experimental contexts represented in the cited literature and are intended as qualitative summaries rather than formal certainty-of-evidence grades. “Human critical illness” refers to observational or interventional studies conducted in critically ill populations; “preclinical” includes animal, ex vivo, and in vitro mechanistic studies. Microbial indole metabolism is presented separately from the predominantly host-derived kynurenine/IDO pathway, and bile-acid interpretation requires distinction among primary, conjugated, and secondary species.

**Figure 2:**
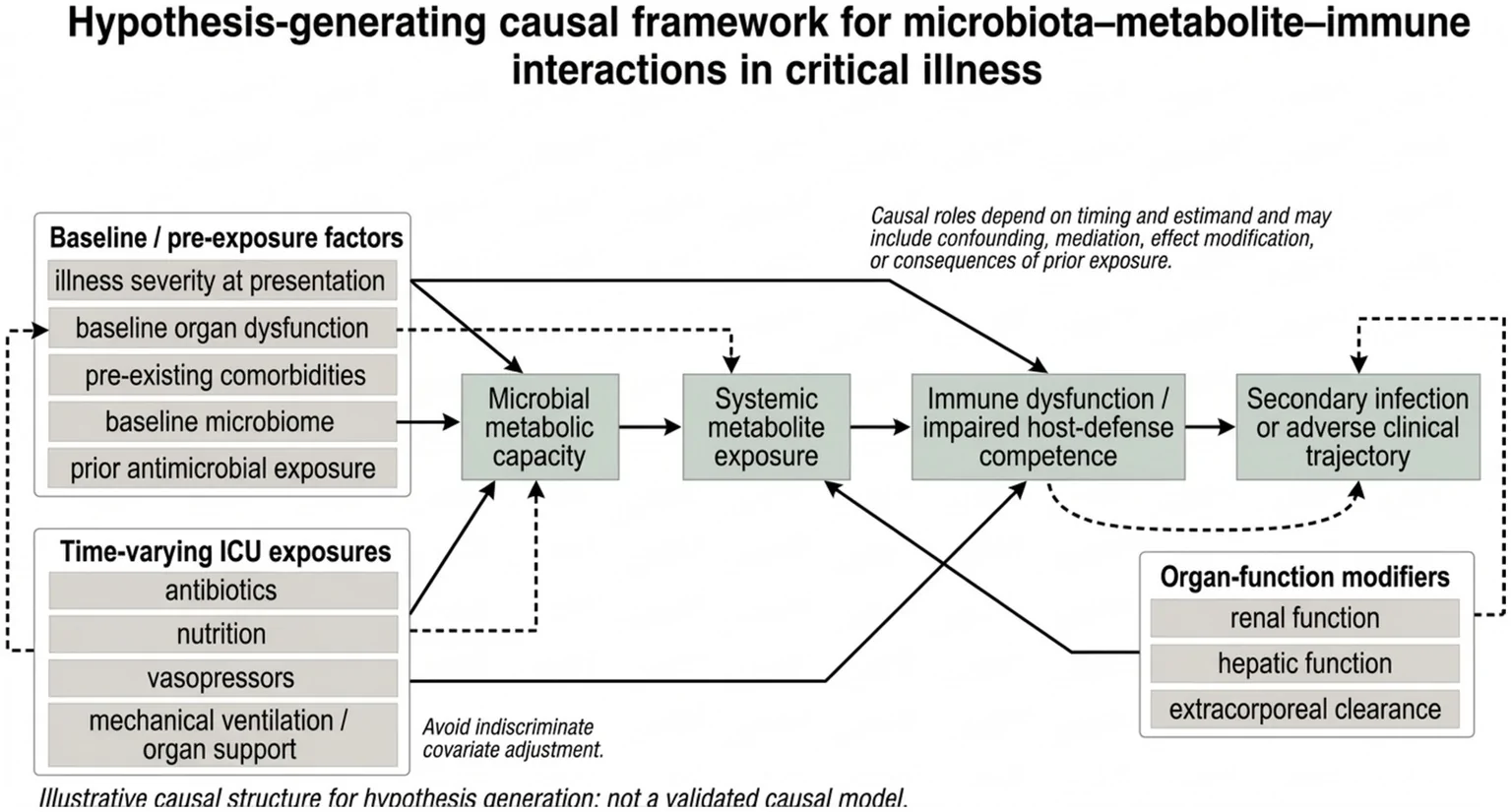
Hypothesis-generating causal framework for microbiota–metabolite–immune interactions in critical illness. The central hypothesized pathway links microbial metabolic capacity to systemic metabolite exposure, immune dysfunction or impaired host-defense competence, and subsequent secondary infection or adverse clinical trajectories. Baseline host factors, time-varying ICU exposures, and organ-function modifiers may occupy different causal roles according to timing and the estimand of interest, including confounding, mediation, effect modification, or consequences of prior exposure. Solid arrows indicate the primary hypothesized pathway and selected determinants of exposure, whereas dashed arrows indicate potential time-dependent feedback relationships. The framework is illustrative and intended to guide causal hypothesis specification rather than represent a validated causal model.

## 7 What human critical-care studies currently establish

### 7.1 Human associations are informative, but causal mediation remains uncommon

Several human studies have reported associations of gut dysbiosis and metabolomic disturbance with mortality, secondary infection, and prolonged organ support, although relatively few longitudinal investigations have examined microbiota, metabolite exposure, and immune phenotype simultaneously within the same patients. Fecal metabolite profiling has identified metabolic patterns associated with increased 30-day mortality risk in critically ill patients (de Porto et al., 2025). Integrated intestinal microbiome and urinary metabolome data have been used to predict secondary infection (Linz et al., 2026), while host–microbe multiomic profiling has associated mortality with neutrophil degranulation, reduced T-cell signaling, and systemic inflammatory programs (Spottiswoode et al., 2026). These approaches extend beyond taxonomic descriptions by linking microbial ecology with metabolic and host-response phenotypes, although they do not, on their own, establish a causal microbiota–metabolite–immune pathway.

Prediction and association are therefore most appropriately considered separately from mediation. A circulating or fecal metabolite may improve prognostic discrimination because it reflects renal dysfunction, hepatic impairment, nutritional substrate, antimicrobial exposure, or tissue hypoxia rather than because the metabolite itself mediates immune dysfunction. Many available datasets are single-center, lack pre-illness baselines, rely on limited sampling time points, or measure stool and circulating metabolites asynchronously. Broad-spectrum antibiotics, nutrition, vasopressors, and organ-support interventions are also time-varying exposures that both alter the microbiome and respond to illness severity. Mortality analyses may be further complicated by competing risks and informative missingness because patients who die early, recover rapidly, receive prolonged antibiotics, or stop producing stool contribute systematically different sampling opportunities.

Clinical immune phenotyping introduces an additional layer of uncertainty. mHLA-DR, lymphocyte counts, checkpoint expression, ex vivo cytokine production, antimicrobial killing, and secondary infection each capture different dimensions of immune competence. Persistent low mHLA-DR has been associated with nosocomial infection in adult septic shock (Landelle et al., 2010), but no single immune marker is likely to capture the functional immune state of all critically ill patients. More informative human studies would therefore benefit from repeated, multidimensional immune measurements linked to microbiologically adjudicated infection and synchronized microbiome–metabolite sampling. Such designs may provide a stronger basis for distinguishing prognostic association from a plausible microbiota–metabolite–immune causal pathway. Representative human studies are summarized individually in Table 2, including study population and sample size, biospecimen and sampling timing, analytical approach, principal findings, analytical or adjustment strategy, major limitations, and the level of inference that can reasonably be supported.

**Table 2:** Representative human studies linking microbiome, metabolomic, and immune disturbances with critical-illness trajectories.

Study	Design and population	Biospecimen, timing, and analytical approach	Main finding	Effect estimate/analysis	Major limitation	Inference supported
Stortz et al., 2018	Prospective observational study; 88 critically ill patients with sepsis and 20 healthy controls	Serial blood sampling; absolute lymphocyte counts, mHLA-DR, and soluble PD-L1	Patients developing chronic critical illness showed sustained lymphopenia, reduced mHLA-DR, increased soluble PD-L1, and more secondary infections than those with rapid recovery	Longitudinal comparison across clinical trajectories and secondary-infection status	Immune phenotyping was not integrated with microbiome or metabolite measurements; does not establish microbial or metabolic causality	Immune dysfunction can persist and evolve along distinct post-sepsis trajectories
Lamarche et al., 2018	Prospective observational study; 34 mechanically ventilated ICU patients, 65 specimens	Endotracheal aspirate, gastric aspirate, and stool; median ~3 days for respiratory/gastric samples and 6 days for stool; 16S rRNA gene sequencing	Critical illness was associated with loss of anatomical microbial differentiation; lower respiratory microbial diversity was associated with greater illness severity and hospital mortality	Respiratory Shannon diversity was lower in nonsurvivors (1.348 vs. 1.964; *p* = 0.045)	Small cohort; relatively few stool samples; strong potential for confounding by severity, antibiotics, and ICU exposure	Multicompartment microbial dysbiosis accompanies severe critical illness and adverse outcome, but does not establish a metabolite-mediated mechanism
Ojima et al., 2022	Prospective longitudinal ICU cohort; 71 mechanically ventilated patients	Serial fecal samples on days 1–2, 3–4, 5–7, 8–14, and thereafter; 16S rRNA gene deep sequencing	Gut microbial diversity declined during ICU stay; microbiome changes were associated with antibiotic exposure, illness severity, and mortality	Extreme Bacteroidetes/Firmicutes imbalance within 7 days was associated with mortality: OR 5.54, 95% CI 1.39–22.18	Single-center observational study; antibiotic exposure and disease severity are time-varying and tightly linked to microbiome change	ICU dysbiosis is dynamic and clinically associated, but directionality and causal mediation remain unresolved
Zhou et al., 2023	Prospective study; 62 mechanically ventilated critically ill patients; 27 died within 28 days	Paired BALF and stool within 48 h of intubation and again at day 7; 16S rRNA sequencing; fecal SCFA measurement	Lung and gut microbial features differed according to outcome; several fecal SCFAs were lower in nonsurvivors	Lung diversity: aHR 0.773, 95% CI 0.652–0.916; fecal Enterococcaceae: aHR 1.022, 95% CI 1.008–1.037 for 28-day mortality	Modest sample size; microbiome and metabolite associations remain vulnerable to illness severity, treatment exposure, and reverse causation	Paired gut–lung profiling provides prognostic information but does not establish that altered microbiota or SCFAs causally determine mortality
Ferrario et al., 2016	Retrospective pilot analysis; 20 patients with severe septic shock from the ALBIOS study	Plasma at day 1 and day 7; targeted quantitative LC–MS/MS metabolomics covering amino acids, acylcarnitines, phospholipids, sphingolipids, and related metabolites	Lipid species and kynurenine-related metabolic alterations differed between survivors and nonsurvivors	Elastic-net and cross-validated metabolomic models evaluated 28 and 90-day mortality	Very small exploratory cohort; high-dimensional analysis relative to sample size; microbial origin not addressed	Circulating metabolic profiles are associated with septic-shock outcome but cannot be interpreted as microbiota-specific signals
Kosyakovsky et al., 2022	Single-center observational cohort; 60 ICU patients with sepsis; 28-day mortality 42%	Plasma obtained ≤72 h after ICU admission; gas/liquid chromatography–mass spectrometry measuring 411 metabolites	Lactate, kynurenine, bile-acid species, phenylalanine, indole acetate, and other metabolites were prioritized as mortality-associated features	Seven machine-learning approaches with internal cross-validation; 13 metabolites were highly ranked across models	Small single-center cohort; internal rather than external validation; metabolite source not resolved	Plasma metabolomics captures outcome-associated metabolic networks but does not establish microbial production or causal mediation
Jennaro et al., 2022	Secondary metabolomic analysis of mechanically ventilated, vasopressor-dependent septic-shock patients; 52 placebo-arm patients, with LC–MS/MS data in 47	Baseline serum within 36 h of mechanical ventilation; LC–MS/MS and ^1H-NMR metabolomics	Two acylcarnitines and four amino acids were associated with time to extubation and vasopressor liberation; several amino acids were elevated in chronic critical illness	Competing-risk survival analysis with death as competing event; metabolite associations had subdistribution HR < 1 and FDR < 0.05	Small secondary-analysis cohort; baseline metabolomics only; predominantly host metabolic signals rather than source-resolved microbial metabolites	Host metabolic state is associated with organ-support trajectory, but metabolic alteration may be marker, mediator, or consequence of illness
de Porto et al., 2025	Prospective MICU cohort; 196 patients with non-COVID respiratory failure or shock	Prospectively collected feces; shotgun metagenomic sequencing plus mass-spectrometric quantification of fecal microbiota-associated metabolites	Initial microbiome composition alone was not independently associated with 30-day mortality, whereas a 13-metabolite metabolic dysbiosis score was associated with mortality	Multivariable prognostic modelling accounting for established confounders	Single-center cohort; stool timing depends on stool availability; fecal concentrations do not directly quantify systemic exposure or causal mediation	Fecal metabolic function may carry prognostic information beyond taxonomic composition, while providing an important example of discordance between microbiome composition and outcome
Linz et al., 2026	Two mechanically ventilated ICU cohorts; core dataset *n* = 88; detailed UHC integrated-analysis subset *n* = 55	Baseline intestinal/respiratory microbiome and urine collected within 48 h of ICU admission or intubation; 16S rRNA sequencing and urinary NMR metabolomics	Secondary infection was associated with lower intestinal diversity and distinct urinary metabolomic features; integrating clinical, microbiome, and metabolome data modestly improved prediction	Core integrated model AUC ~ 0.75 versus 0.64 for clinical data alone; Shannon diversity and selected urinary NMR features remained informative in multivariable models	Modest cohorts; prediction does not establish mediation; microbial and urinary metabolite features may reflect antibiotic exposure, severity, and organ function	Synchronized multi-compartment profiling can improve risk stratification for secondary infection, but causal microbiota–metabolite–immune mediation remains unproven
Spottiswoode et al., 2026	Prospective multicenter critical-care cohort; 321 critically ill adults analyzed; microbiologically confirmed sepsis *n* = 130	Whole blood and plasma obtained in the ED or within 24 h of ICU admission; RNA sequencing, plasma protein measurements, and plasma cell-free DNA metagenomics	Sepsis mortality was associated with greater neutrophil-degranulation signaling, reduced T-cell signaling, increased IL-8, and altered microbial signals	Differential-expression analysis and bagged support-vector-machine classifiers with 10-fold cross-validation	Blood/plasma microbial signals are not equivalent to intestinal microbiome measurements; cross-sectional early sampling limits causal ordering	Host and microbial signals jointly associate with sepsis mortality, supporting integrated profiling rather than a simple gut-metabolite causal pathway
Chou et al., 2021	Single-center prospective cohort; 95 mechanically ventilated patients with sepsis; 129 elective-coronary-angiography controls	Plasma within 24 h of ICU admission; TMAO measurement; nutritional and antibiotic exposures recorded	Septic patients had lower TMAO than cardiovascular controls; low TMAO was associated with poorer nutrition and greater prior antibiotic exposure	Lowest versus highest TMAO tertile: HR 2.51, 95% CI 1.21–5.24 for non-CV death; association attenuated after adjustment for nutritional status	Single center; circulating TMAO strongly influenced by diet, antibiotics, renal function, hepatic conversion, and nutritional status	Circulating TMAO cannot be interpreted as a direct measure of intestinal microbial activity or as uniformly harmful in sepsis
Xu et al., 2026	Prospective single-center study; 87 analyzed patients with sepsis and 13 healthy controls*	Serum obtained within 24 h of ICU admission; 15 bile acids quantified by LC–MS/MS	Secondary bile acids were reduced in sepsis; nonsurvivors had higher TCA/TDCA and lower UDCA	UDCA and 28-day mortality: univariate Cox HR 0.990, *p* = 0.042; ROC AUC 0.643	Single center; serum bile acids reflect hepatic synthesis/conjugation, cholestasis, nutrition, and microbial transformation simultaneously	Bile-acid speciation is associated with sepsis severity and outcome but cannot establish microbial source attribution
Beloborodova et al., 2018	Clinical cohort of community-acquired pneumonia; septic shock *n* = 20 and non-shock *n* = 21	Blood measurements at ICU admission and the following day; aromatic microbial metabolites, catecholamine metabolites, lactate, NT-proBNP, iNOS, and PCT	Phenyllactic and hydroxyphenyl acids were higher in septic shock and correlated with lactate, catecholamine metabolites, and cardiovascular-stress markers	Sum of three aromatic metabolites correlated with lactate (*r* = 0.67 at admission; *r* = 0.73 next day) and other shock-related measures	Very small cohort; correlations cannot separate microbial production from shock physiology, catecholamine exposure, organ dysfunction, or clearance	Aromatic metabolic disturbance accompanies severe septic-shock physiology but does not establish a causal microbial-metabolite pathway

This table is intended to illustrate the heterogeneity and inferential limits of representative human studies rather than to provide formal evidence grading. Effect estimates are shown where they were central to the original analysis and readily interpretable. Associations or predictive performance do not establish microbial source attribution, metabolite-mediated causality, or therapeutic direction.

### 7.2 Metabolite panels better reflect network-level biology than single-molecule models

The metabolome is better understood as an interconnected network than as a collection of independent biomarkers. In sepsis, mortality-associated signatures have included lactate, kynurenine, phenylalanine, conjugated and secondary bile-acid species, indole acetate, and metabolites related to mitochondrial stress (Kosyakovsky et al., 2022; Rogers et al., 2024). These pathways are biologically interdependent: antibiotics may reduce indole and SCFA production while altering bile-acid transformation; kidney dysfunction can increase retention of aromatic co-metabolites and TMAO; and hepatic dysfunction can modify bile-acid conjugation, aromatic-amino-acid metabolism, and lactate clearance. Consequently, an apparent association with a single metabolite may partly reflect variation arising from several interconnected microbial, host, and organ-clearance processes.

Multimetabolite panels may therefore offer a more informative representation of critical-illness biology, provided that their mechanistic interpretability is preserved. Candidate panels could include complementary features representing microbial fermentation, barrier-associated indole metabolism, host kynurenine/IDO activity, bile-acid transformation, mitochondrial stress, aromatic co-metabolite retention, and renal or hepatic clearance. Where feasible, these features may be most informative when integrated with metagenomic pathway abundance and functional immune measurements rather than considered as isolated predictors. Predictive performance alone is unlikely to be sufficient for clinical interpretation; model calibration, external validation, transparent handling of missing data, and robustness across major time-varying exposures such as nutrition, antibiotics, and organ-support strategies would also need to be considered before such panels can be regarded as clinically informative.

### 7.3 Current intervention data do not yet test immune-state–adapted metabolite therapy

Probiotics, synbiotics, and fermentable substrates are among the most extensively studied microbiota-directed interventions in critically ill patients. A large randomized trial found no reduction in ventilator-associated pneumonia with *Lactobacillus rhamnosus* GG in critically ill patients (Johnstone et al., 2021). A smaller randomized study reported that synbiotic therapy altered the gut microbiota and was associated with lower rates of enteritis and ventilator-associated pneumonia in patients with sepsis (Shimizu et al., 2018), highlighting substantial heterogeneity in study populations, microbial products, background care, and clinical endpoints. A phase 2 trial of inulin in ICU patients with sepsis targeted gut pathogen colonization and infection-related outcomes (Park et al., 2025). However, these studies were not primarily designed to stratify patients by immune state, quantify the intended metabolite exposure, or determine whether observed clinical effects were mediated through a defined microbiota–metabolite–immune pathway.

Taken together, the available evidence does not readily support a simple class-wide judgment that microbiome-directed interventions are uniformly effective or ineffective. Their effects are likely to vary according to strain or microbial community, substrate availability, baseline ecology, antibiotic exposure, colonization resistance, delivery timing, organ function, and host immune state. Safety considerations are similarly context dependent: live microorganisms may warrant particular caution in patients with severe barrier disruption or profound immune dysfunction, whereas purified postbiotics avoid replication but may not reproduce the spatial distribution, duration, or local concentration achieved through endogenous microbial production. Future intervention studies would benefit from clearly defining the intended metabolic output, relevant receptor or host pathway, exposure kinetics, immune context, and host-defense endpoints, while also examining whether changes in the proposed metabolite pathway mediate the observed biological or clinical effects.

### 7.4 Generalizable biology and diagnosis-specific modifiers

A state-centered framework may help identify biological processes shared across sepsis, trauma, burns, pancreatitis, ARDS, and postoperative critical illness, although meaningful generalization also depends on recognition of diagnosis-specific mechanisms. Sterile tissue injury can generate host-derived succinate and lactate in the absence of direct microbial invasion; abdominal sepsis may involve enteric microbial translocation or inadequate source control; pneumonia places substantial antimicrobial demands on alveolar macrophages and neutrophils; and major burns are characterized by prolonged hypermetabolism, barrier disruption, repeated procedures, and high antimicrobial exposure. Gut-associated bacteria have also been reported to enrich the lung microbiome in sepsis and ARDS, further illustrating the potential for cross-compartment microbial interactions during critical illness (Dickson et al., 2016). These settings may therefore give rise to similar circulating metabolite patterns through biologically distinct pathways.

Sepsis-associated liver injury may further modify gut–liver interactions, host metabolic processing, and metabolite clearance, while sepsis-associated acute kidney injury may alter microbiota–host relationships through changes in renal clearance and the broader gut–kidney axis (Moura and Siqueira, 2025; Zhang et al., 2026). These organ-specific interactions may further complicate interpretation of circulating microbial–host co-metabolites.

Pathogen identity may further modify the functional significance of metabolite signaling. Butyrate can impair early neutrophil control of *Pseudomonas aeruginosa* yet enhance macrophage antimicrobial activity in experimental leptospirosis (Dang et al., 2023; Chen et al., 2024). Fungal secondary infection, recurrent Gram-negative bacteremia, and viral reactivation may likewise arise from different combinations of innate, adaptive, and barrier dysfunction. Interpretation of metabolite–immune relationships may therefore be more informative when infectious source and pathogen class are considered explicitly. Similarly, sterile inflammatory models are unlikely to be fully interchangeable with sepsis because they do not reproduce the dynamic contribution of viable-pathogen burden and antimicrobial clearance.

Host factors introduce an additional layer of heterogeneity. Age, frailty, obesity, diabetes, chronic kidney disease, cirrhosis, malignancy, and pre-existing immunosuppression can alter baseline microbial ecology, organ clearance, mitochondrial reserve, receptor expression, and immune-cell composition. In experimental metabolic inflammation, the anti-inflammatory macrophage program associated with SUCNR1 signaling was attenuated in obesity (Keiran et al., 2019), illustrating how host state can modify receptor-dependent effects. Spermidine-dependent autophagy may also be particularly relevant to age-associated decline in CD8 T-cell function (Alsaleh et al., 2020). Collectively, these observations favor a stratified interpretation of critical-illness metabolomic phenotypes rather than the assumption of a single universal pattern.

One practical approach may be to combine a shared core design with diagnosis-specific modules. Across cohorts, synchronized measurements of microbiome composition or function, metabolite exposure, immune phenotype, treatment exposures, and organ function could provide a common analytical backbone. Additional modules could capture source-control adequacy, pathogen class, burn severity, trauma burden, transplant status, or other diagnosis-specific factors. Replication across conditions may be most informative when based on consistency in temporal pattern and mechanistic direction rather than on recurrence of an isolated association with mortality.

## 8 Therapeutic implications: separating standard critical-care practice from microbiome-directed hypotheses

### 8.1 Distinguishing standard critical-care practice from microbiome-specific therapy

Several aspects of routine critical-care management can influence microbial ecology and metabolite availability, including antimicrobial exposure, enteral nutrition, fasting, acid suppression, vasopressors, and organ-support interventions. However, these practices are more appropriately viewed as components of standard critical care rather than as microbiome-directed therapies simply because they modify the microbiota–metabolite–host axis.

Antimicrobial selection, spectrum, duration, and de-escalation are primarily guided by pathogen risk, source control, microbiological data, and clinical response. Potential effects on anaerobic community structure or microbial metabolic function are therefore best considered secondary research questions and should not take precedence over timely and adequate treatment of infection. From a microbiome perspective, a more relevant question is whether anti-infective strategies with comparable antimicrobial efficacy differ in their ecological or metabolic consequences and whether such differences contribute to downstream immune or clinical outcomes.

Nutritional support is likewise most appropriately guided by established critical-care principles rather than by microbiome hypotheses alone. In hemodynamically stabilized critically ill patients who cannot maintain adequate oral intake, current critical-care nutrition guidance favors early, progressive enteral nutrition, generally initiated within 48 h, rather than prolonged fasting or routine early parenteral nutrition (Singer et al., 2023). In sepsis, advancement of enteral feeding is generally guided by clinical stability and gastrointestinal tolerance. From a microbiome perspective, substrate composition remains biologically relevant because fermentable carbohydrates and selected soluble fibers can influence microbial fermentation and SCFA production; however, the optimal substrate, dose, timing, and target population remain uncertain. Fiber supplementation is therefore better regarded as an area of ongoing investigation rather than as an established microbiome-restorative intervention.

The same distinction applies to immunonutrition and other supportive strategies. Formulas enriched with arginine, glutamine, omega-3 fatty acids, nucleotides, or antioxidant substrates may alter immune and metabolic responses, but heterogeneous ICU evidence does not establish that these interventions restore microbiota-derived metabolites or improve host defense through a defined microbiota–metabolite pathway. Their use is therefore more appropriately based on established nutritional or critical-care indications than on an assumed microbiome mechanism.

### 8.2 Microbiome and metabolite-directed interventions remain investigational

An *immune-state–adapted research framework* may provide a useful structure for organizing future microbiota–metabolite intervention studies, although the current evidence base remains insufficient to support prescribing specific metabolite strategies for individual patients or assigning patients reliably to therapeutic strata. The examples below are therefore best viewed as hypothesis-generating research directions rather than as treatment recommendations.

During *hyperinflammatory states*, candidate pathways for investigation may include barrier-supportive indole signaling, compartment-targeted SCFA exposure, receptor-selective modulation of TGR5 or GPR81, and attenuation of excessive succinate/HIF-1α signaling. Studies in this setting would benefit from characterizing the inflammatory phenotype alongside measures of antimicrobial competence. Relevant outcomes could include organ injury, endothelial dysfunction, microbial clearance, pathogen burden, and the subsequent evolution of immune function. A reduction in inflammatory signaling alone is unlikely to provide sufficient evidence of benefit if host-defense function is simultaneously impaired.

During *states characterized predominantly by immunosuppression*, further suppression of inflammatory signaling may be less desirable when antimicrobial competence is already compromised. Candidate approaches may therefore be more meaningfully evaluated according to their effects on phagocytosis, antigen presentation, mitochondrial function, lymphocyte competence, or colonization resistance rather than on reductions in cytokine concentrations alone. Microbial indole pathways with phagocytosis or bactericidal-enhancing effects, restoration of pyruvate oxidation, and ecological strategies that reinforce colonization resistance represent mechanistically plausible research directions. Potential enrichment features may include persistently reduced mHLA-DR relative to a prespecified assay-specific threshold or failure of mHLA-DR recovery over serial measurements, impaired ex vivo immune responsiveness, lymphocyte dysfunction, viral reactivation, or microbiologically adjudicated secondary infection; however, these features have not been validated as standalone criteria for therapeutic assignment, and no single marker is likely to capture the full spectrum of immunosuppression.

During *recovery or persistent inflammation–immunosuppression–catabolism*, different biological priorities may emerge, including epithelial repair, mitochondrial quality control, efferocytosis, preservation of muscle metabolism, and restoration of adaptive immune function. SCFAs and polyamines may warrant investigation in this context because of their effects on barrier integrity, autophagy, and reparative immune programs, although their consequences are likely to remain dependent on nutritional substrate, organ function, residual pathogen burden, and concurrent rehabilitation or metabolic support. Restoration of ecological function may ultimately depend on coordinated changes in microbial substrate availability, community structure, and host metabolic capacity rather than on supplementation with a single metabolite.

Taken together, these examples are best regarded as *testable, hypothesis-generating research concepts rather than validated therapeutic strata or treatment recommendations*. The proposed enrichment features, candidate interventions, pharmacodynamic endpoints, and safety or futility criteria are illustrative and have not been prospectively validated as a combined decision framework in critically ill patients. Future trials would therefore benefit from prospective immune phenotyping, synchronized metabolite measurements, predefined host-defense endpoints, demonstration of pathway-specific target engagement, and evidence that the intended biological effect can be achieved without compromising antimicrobial competence before *immune-state–adapted metabolite interventions* are considered for broader clinical evaluation. The resulting *hypothesis-generating intervention framework*, including candidate enrichment features, pharmacodynamic requirements, and safety or futility signals, is summarized in Table 3.

**Table 3:** Hypothesis-generating framework for testing immune-state–adapted metabolite interventions in critical illness.

Proposed immune state	Candidate enrichment features	Candidate intervention concept	Required pharmacodynamic evidence	Safety or futility signal
Hyperinflammatory state with preserved host defense	Elevated inflammatory or endothelial markers together with serial mHLA-DR measurements remaining above a prespecified, assay-specific reference threshold and/or preserved ex vivo immune responsiveness	Locally acting barrier-supportive indole or SCFA approaches; selective modulation of succinate-, TGR5-, or GPR81-related pathways	Demonstrated target engagement, reduced tissue or endothelial injury, and preserved antimicrobial killing	Rising pathogen burden, new secondary infection, or deterioration in functional immune competence
Predominant immunosuppression	Persistently reduced mHLA-DR relative to a prespecified, assay-specific threshold or failure of mHLA-DR recovery over serial measurements, impaired ex vivo stimulation responses, lymphocyte dysfunction, viral reactivation, or microbiologically adjudicated secondary infection	Bactericidal or phagocytosis-enhancing indole pathways, ecological restoration, or metabolic reactivation	Improved phagocytosis, oxidative burst, antigen presentation, or T-cell function	Inflammatory rebound with organ injury or failure to improve antimicrobial competence
Recovery or persistent inflammation–immunosuppression–catabolism	Persistent low-grade inflammation, catabolism, barrier dysfunction, weakness, or prolonged organ-support dependence	Nutritional substrate support, targeted postbiotics, or ecological strategies favoring SCFA/polyamine-associated repair pathways	Improved barrier function, mitochondrial fitness, efferocytosis, muscle function, or adaptive immune recovery	Metabolite accumulation during organ failure, recurrent infection, intolerance, or absence of functional benefit
Unclassified or rapidly changing immune state	Discordant biomarkers, new shock, new infection, or rapidly changing organ function	Serial phenotyping and supportive care before pathway-specific intervention is considered	Reproducible immune-state classification across repeated measurements	Any pathway-specific intervention based solely on a single metabolite concentration or isolated biomarker

This table is intended to generate testable research hypotheses and does not define validated patient strata, biomarker thresholds, or treatment recommendations. The proposed enrichment features, interventions, pharmacodynamic endpoints, and futility signals require prospective validation. mHLA-DR thresholds are not universally standardized across assays, laboratories, patient populations, or sampling time points; mHLA-DR should therefore be interpreted using pre-specified assay-specific thresholds and longitudinal trajectories rather than a single universal cutoff. No isolated biomarker is sufficient to assign a patient to a therapeutic stratum.

### 8.3 Receptor and compartment-selective strategies as future research directions

Whole-metabolite supplementation can engage multiple receptors, transporters, and metabolic pathways across different tissues. More selective strategies for investigation may include non-absorbed or locally retained formulations, engineered microorganisms with controlled metabolic output, microbial-enzyme inhibitors, receptor-selective or biased agonists, and modulation of metabolite transport. Inhibition of microbial CutC/D provides a useful proof of concept that a defined microbial enzymatic pathway can be suppressed without directly killing the producing organisms (Roberts et al., 2018), although the relevance of this strategy to critically ill patients remains uncertain. Conversely, engineered microbial production of a defined metabolite could, in principle, help localize exposure to the intestinal compartment and reduce unintended systemic effects, although this approach would require careful evaluation of output stability, colonization behavior, antimicrobial susceptibility, and biosafety.

Greater selectivity does not necessarily remove the importance of source and immune-state stratification. Receptor abundance and downstream signaling may vary across cell types and immune states, while receptor-independent effects such as HDAC inhibition, substrate utilization, redox modulation, or protein acylation may become relevant at different concentrations. Pharmacodynamic studies would therefore benefit from demonstrating target engagement within the relevant anatomical compartment and cell population rather than relying solely on circulating metabolite concentrations. Where feasible, it would also be important to determine whether such target engagement produces the intended functional effect without compromising antimicrobial competence.

## 9 A clinical research framework for metabolite-guided immune modulation

### 9.1 Cohort design and sampling

Future discovery cohorts would benefit from early enrollment and synchronized longitudinal sampling. Where feasible, research sampling may begin as early as possible after ICU admission and before major treatment exposures, provided that this does not interfere with clinical care. Importantly, research sampling must never delay urgent antimicrobials, hemodynamic stabilization, source control, respiratory support, or any other clinically indicated treatment. Subsequent sampling could be scheduled at prespecified intervals such as days 1, 3, 7, 14, and 28, together with clinically meaningful transitions including secondary infection, liberation from organ support, recurrence of shock, or major antimicrobial changes. Because stool availability is often inconsistent in critically ill patients, rectal swabs, plasma, urine, and whole blood may provide a more reliable longitudinal core, with stool and, when clinically obtained, bronchoalveolar or intestinal samples collected in nested subgroups. Antibiotic exposure, enteral feeding dose and composition, vasopressor dose, sedation, acid suppression, bowel function, renal replacement therapy, and hepatic dysfunction would be more informative if recorded as time-varying exposures rather than treated only as baseline covariates.

The choice of microbiome profiling method is best guided by the underlying biological question. When microbial metabolic capacity or pathway activity is central, shotgun metagenomics—and, where feasible, metatranscriptomics—can provide greater functional resolution than 16S rRNA profiling alone. Targeted quantitative metabolomics may be complemented by untargeted discovery where appropriate and supported by internal standards and quality-control procedures. Plasma, urine, and fecal metabolite concentrations are likely to be most informative when interpreted alongside renal function, bilirubin, albumin, dialysis exposure, nutritional substrate, and relevant medications. For metabolites with substantial shared host and microbial production, including succinate, lactate, and selected aromatic pathways, stable-isotope tracing or isotope-enabled ex vivo microbial culture may help strengthen source attribution.

Immune phenotyping would likewise benefit from a longitudinal and functional approach rather than reliance on static cell counts alone. A practical core could include mHLA-DR, absolute lymphocyte count, T-cell activation and exhaustion markers, neutrophil maturation and oxidative burst, monocyte cytokine production after standardized stimulation, and circulating inflammatory mediators. Secondary infections are likely to be most informative when adjudicated with explicit onset timing and microbiological documentation whenever possible. Clinical outcomes may include organ-support-free days, persistent organ dysfunction, ICU-acquired infection, discharge disposition, muscle or functional status, and longer-term survival. A central design principle is synchronization: microbiome, metabolite, immune, treatment-exposure, and organ-function measurements are most useful when aligned closely enough in time to support temporal and mechanistic inference.

### 9.2 Causal analysis

Longitudinal analyses would benefit from evaluating temporal ordering across the proposed microbiota–metabolite–immune pathway: whether changes in microbial metabolic capacity precede changes in metabolite exposure, whether metabolite changes precede alterations in immune function, and whether immune trajectories mediate subsequent clinical outcomes. At the same time, causal analysis is most informative when the specific estimand and the assumed temporal causal structure are defined explicitly rather than when all available clinical variables are adjusted for indiscriminately.

The role of a given variable may change according to the scientific question and time point. For example, renal dysfunction may act as a baseline confounder if it predates the measured metabolite exposure, as a mediator if metabolite-associated processes contribute to subsequent kidney injury, as an effect modifier if impaired clearance alters exposure–response relationships, or as a consequence of prior treatment or metabolite exposure. Similar considerations apply to antibiotics, nutrition, vasopressors, hepatic dysfunction, and organ-support interventions. Broad adjustment for such variables may therefore, in some settings, introduce overadjustment or collider bias.

A causal diagram can be particularly useful for making these assumptions explicit in each primary analysis. One illustrative structure is: microbial metabolic capacity → systemic metabolite exposure → immune dysfunction → secondary infection. Baseline illness severity, pre-existing organ dysfunction, and prior antimicrobial exposure may confound parts of this pathway, whereas subsequent antibiotics, nutrition, vasopressors, renal or hepatic dysfunction, and organ support may function as time-varying confounders, mediators, or consequences of prior exposure depending on the estimand.

Mediation models may help quantify pathway-specific effects when temporal ordering, sampling density, and assumptions about mediator–outcome confounding are sufficiently supported. Marginal structural models may be useful when time-varying confounders are themselves affected by prior exposure. Competing-risk methods may be appropriate for outcomes such as secondary infection or liberation from organ support when death precludes the event, while joint or shared-parameter models may help address informative dropout and irregular longitudinal sampling.

No single statistical model is likely to be sufficient to establish a causal microbiota–metabolite–immune pathway. Stronger inference is more likely to emerge from triangulation across complementary sources of evidence. A candidate pathway becomes more credible when metagenomic functional capacity, compartment-specific metabolite exposure, receptor or pathway activation, and immune phenotype change coherently over time; when associations persist after appropriately specified adjustment for renal and hepatic clearance; when relevant microbial isolates or communities generate the candidate metabolite; and when gnotobiotic transfer, isotope tracing, or receptor/pathway perturbation reproduces the proposed biological effect. Mendelian randomization may provide complementary evidence for relatively stable host determinants, although it is unlikely to resolve rapidly changing ICU exposures or short-term microbial–metabolic dynamics on its own.

### 9.3 Adaptive trials

Early-phase trials would ideally establish pharmacokinetics, target engagement, ecological effects, and safety before moving toward definitive evaluation of clinical efficacy. A biomarker-enriched platform could prospectively classify patients into hyperinflammatory, immunosuppressed, or recovery-associated immune states using prespecified immune and metabolic criteria. Within these strata, pathway-matched interventions could be evaluated using adaptive or response-adaptive trial designs where appropriate. A shared core outcome set may facilitate comparison across immune states while still allowing state-specific biological and clinical endpoints.

Safety monitoring would also benefit from extending beyond conventional adverse-event reporting to include impaired microbial clearance, bloodstream infection related to an administered organism, excessive inflammatory rebound, metabolite accumulation during renal or hepatic dysfunction, and unexpected organ-specific toxicity. Trials may also be more informative if they assess whether an intervention improves functional immune competence and host-defense trajectory rather than merely shifting a circulating metabolite concentration toward a predefined range. Table 3 summarizes the proposed intervention logic, and Table 4 outlines priority experimental questions that may help guide broader evaluation of immune-state–adapted metabolite strategies.

**Table 4:** Priority research questions and key design features for causal and translational studies.

Priority question	Key design feature	Evidence that would strengthen inference
Is circulating succinate or lactate substantially microbial in origin?	Paired compartment sampling, isotope tracing, metagenomic functional capacity, and organ-clearance measurements	Changes in microbial metabolic capacity precede or parallel appearance of labeled metabolite in systemic compartments, with host contribution quantified
Does metabolite change mediate an immune trajectory?	Serial microbiome, metabolome, and functional immunophenotyping with temporally aligned longitudinal analysis	Metabolite change occurs between microbial-pathway change and subsequent immune-function change and remains consistent after adjustment for major time-varying exposures
Does cytokine suppression represent resolution or immune dysfunction?	Concurrent measures of tissue injury, pathogen burden, phagocytosis, oxidative burst, and secondary infection	Reduced inflammatory injury accompanied by preserved or improved antimicrobial clearance rather than viable-pathogen persistence
Which patients may benefit from an SCFA or indole-directed intervention?	Immune-state-enriched trial with molecule-specific pharmacokinetics and pharmacodynamic target-engagement measures	Benefit or biological response is concentrated in a prespecified exposure/immune-state subgroup with demonstrated pathway engagement
To what extent do organ dysfunction and clearance determine aromatic co-metabolites and TMAO?	Free and total analyte measurements, renal and hepatic function, dialysis timing/modality, albumin, nutritional substrate, and relevant medications	Associations persist after mechanistically informed production–clearance modeling and source triangulation
Can microbiota-directed interventions be delivered safely during immunosuppression?	Product-level genomic safety assessment, bloodstream surveillance, colonization tracking, metabolite measurements, and antimicrobial-resistance monitoring	Intended ecological or metabolite engagement occurs without microbial translocation, treatment-related infection, or evidence of resistance transfer

## 10 Key uncertainties and limitations

Several conceptual and methodological uncertainties remain important when interpreting evidence in this field. First, the term “microbiota-derived” may at times be applied too broadly. Succinate, lactate, polyamines, and several aromatic intermediates can arise substantially from host metabolism, and circulating concentrations alone are unlikely to establish microbial origin. Second, stool represents a distal ecological compartment and may not fully reflect metabolite production in the small intestine, mucus-associated communities, or other mucosal niches. Third, receptor expression and downstream signaling vary across species, tissues, and immune states, and metabolite concentrations used in experimental models may not always correspond to physiologically achievable or clinically tolerable exposures in critically ill patients. Fourth, antibiotics, nutrition, renal dysfunction, hepatic dysfunction, and organ-support interventions can modify metabolite production, transformation, and clearance and are therefore better considered as biological components of the pathway rather than as statistical confounders alone.

Fifth, many recent mechanistic studies report beneficial or harmful effects in individual sepsis, endotoxemia, ischemia–reperfusion, or lung-injury models without independent replication across pathogens, models, or laboratories. Such findings remain valuable for hypothesis generation, although their interpretation is strengthened when considered alongside more established and reproducible physiological evidence. Sixth, critical illness and sepsis are themselves highly heterogeneous with respect to pathogen, infection source, age, comorbidity, organ dysfunction, treatment exposure, and baseline microbial ecology. A metabolite effect observed in sterile endotoxemia may therefore differ substantially from that seen during polymicrobial infection or persistent viable-pathogen burden. Seventh, immune suppression lacks a universally accepted bedside definition, while secondary infection may reflect not only host-defense competence but also device exposure, antimicrobial treatment, source control, and healthcare-associated risk.

Finally, the immune states used throughout this review are intended as clinical heuristics rather than as discrete or mutually exclusive biological categories. Hyperinflammation, immune suppression, metabolic dysfunction, and tissue repair can coexist and evolve dynamically over time, and patients may transition in either direction following secondary infection, surgery, procedures, or changes in organ function. The value of a state-aware framework therefore lies in improving temporal interpretation, biomarker enrichment, and study design without implying a rigid sequence across heterogeneous critical-illness trajectories.

## 11 Conclusion

Microbial metabolites and microbial–host co-metabolites are plausible regulators of the microbiota–metabolite–host immune axis during critical illness, although their effects appear to be highly context dependent rather than intrinsically beneficial or harmful. SCFAs, indoles, bile acids, succinate, lactate, TMAO, polyamines, and aromatic co-metabolites can influence epithelial barrier integrity, immune metabolism, antimicrobial defense, lymphocyte function, endothelial responses, and tissue repair through overlapping receptor-mediated, mitochondrial, metabolic, and epigenetic pathways. The functional consequences of these signals are likely to be shaped by their source, magnitude and duration of exposure, anatomical compartment, prevailing immune state, pathogen burden, and organ function. Accordingly, the same metabolite-associated pathway may limit collateral tissue injury in one setting while compromising antimicrobial competence or favoring pathogen persistence in another.

Available human studies have reported associations between dysbiosis or metabolomic disturbance and adverse clinical trajectories, including secondary infection, prolonged organ support, and mortality; however, they rarely establish microbial source attribution, temporal mediation, or causal effects on immune function. Current evidence therefore does not yet provide a sufficient basis for assuming that modifying a circulating metabolite concentration will improve clinical outcomes, nor that a circulating metabolite necessarily reflects intestinal microbial activity. Further progress is likely to depend on synchronized longitudinal microbiome, metabolome, immune-function, treatment-exposure, and organ-function measurements; source-resolved approaches such as isotope tracing and microbial functional analysis; and experimental perturbation capable of distinguishing association from causal mediation.

The central translational question is therefore not simply “Which microbial metabolite is beneficial?” but rather “Which metabolic pathway, arising from which source and exposure, and acting within which immune context, is associated with preserved or impaired host defense—and can that pathway be modified without compromising antimicrobial competence?” The source–state–exposure–competence framework proposed here offers a hypothesis-generating structure for addressing this question and may help guide future translational studies in critical illness.
